# Analysis of Secondhand Smoke Exposure and Myopia Among Children Aged 6 to 8 Years in Hong Kong

**DOI:** 10.1001/jamanetworkopen.2023.13006

**Published:** 2023-05-11

**Authors:** Youjuan Zhang, Xiu Juan Zhang, Nan Yuan, Yuzhou Zhang, Yu Meng Wang, Fangyao Tang, Mandy P. Ng, Ian C. K. Wong, Patrick Ip, Ka Wai Kam, Alvin L. Young, Li Jia Chen, Clement C. Tham, Chi Pui Pang, Jason C. Yam

**Affiliations:** 1 Department of Ophthalmology and Visual Sciences, The Chinese University of Hong Kong, Hong Kong SAR, China; 2 The Nethersole School of Nursing, The Chinese University of Hong Kong, Hong Kong SAR, China; 3 Joint Shantou International Eye Center of Shantou University and the Chinese University of Hong Kong, Shantou, China; 4 Kunming Bright Eye Hospital, Kunming, China; 5 Department of Neurobiology, Interdisciplinary Center for Neurosciences, Heidelberg University, Heidelberg, Germany; 6 Centre for Safe Medication Practice and Research, Department of Pharmacology and Pharmacy, Li Ka Shing Faculty of Medicine, The University of Hong Kong, Hong Kong SAR, China; 7 Department of Paediatrics and Adolescent Medicine, University of Hong Kong, Hong Kong SAR, China; 8 Department of Ophthalmology and Visual Sciences, Prince of Wales Hospital, Hong Kong SAR, China; 9 Hong Kong Hub of Paediatric Excellence, The Chinese University of Hong Kong, Hong Kong SAR, China; 10 Hong Kong Eye Hospital, Hong Kong SAR, China; 11 Department of Ophthalmology, Hong Kong Children’s Hospital, Hong Kong SAR, China

## Abstract

**Importance:**

Secondhand smoke (SHS) exposure potentially threatens ocular health; however, its association with myopia is unknown.

**Objective:**

To examine the association between SHS exposure and childhood myopia.

**Design, Setting, and Participants:**

Cross-sectional data from the population-based Hong Kong Children Eye Study were used. Data were collected from March 5, 2015, to September 12, 2021, at The Chinese University of Hong Kong Eye Center. Participants included children aged 6 to 8 years. Secondhand smoke exposure was evaluated using a validated questionnaire. All participants underwent comprehensive ophthalmic and physical examinations.

**Exposure:**

Secondhand smoke exposure.

**Main Outcomes and Measures:**

Generalized estimating equations were constructed to examine the association of SHS exposure with spherical equivalent and axial length; logistic regression models, with myopia rate; and linear regression models, with myopia onset.

**Results:**

A total of 12 630 children (mean [SD] age, 7.37 [0.88] years; 53.2% boys) were included in the analysis. Among the participants, 4092 (32.4%) had SHS exposure. After adjusting for age, sex, parental myopia, body mass index, near-work time, outdoor time, and family income, SHS exposure was associated with greater myopic refraction (β = −0.09 [95% CI, −0.14 to −0.03]) and longer axial length (β = 0.05 [95% CI, 0.02-0.08]). Children with SHS exposure were more likely to develop moderate (odds ratio [OR], 1.30 [95% CI, 1.06-1.59]) and high myopia (OR, 2.64 [95% CI, 1.48-4.69]). The association of SHS exposure with spherical equivalence and axial length was magnified in younger children. For each younger year of a child’s exposure to SHS, SHS exposure was associated with a 0.07-D decrease in spherical equivalence (β = 0.07 [95% CI, 0.01-0.13]) and a 0.05-mm increase in axial length (β = −0.05 [95% CI, −0.08 to −0.01]). Exposure to SHS was associated with an earlier mean (SD) age at onset of myopia (72.8 [0.9] vs 74.6 [0.6] months; *P* = .01). Every increase in SHS exposure in units of 10 cigarettes per day was associated with greater myopic refraction (β = −0.07 [95% CI, −0.11 to −0.02]), axial length (β = 0.04 [95% CI, 0.01-0.06]), and likelihood of developing moderate (OR, 1.23 [95% CI, 1.05-1.44]) and high myopia (OR, 1.75 [95% CI, 1.20-2.56]), and earlier myopia onset (β = −1.30 [95% CI, −2.32 to −0.27]).

**Conclusions and Relevance:**

The findings of this cross-sectional study suggest that SHS exposure was associated with greater myopic refraction, longer axial length, greater likelihood of developing moderate and high myopia, and earlier myopia onset. The larger the quantity of SHS exposure and the younger the child, the more advanced myopia development and progression with which SHS exposure is associated.

## Key Points

**Question:**

Is there an association between secondhand smoke (SHS) exposure and childhood myopia?

**Findings:**

In this cross-sectional study that included 12 630 children aged 6 to 8 years, SHS exposure was associated with greater myopic refraction, longer axial length, greater likelihood of developing moderate and high myopia, and earlier myopia onset. The association was greater with higher exposure and younger age.

**Meaning:**

These findings suggest that eliminating SHS exposure is important for myopia prevention among children, particularly in families with young children.

## Teaser

This cross-sectional study examines the association between secondhand smoke exposure and myopia among children aged 6 to 8 years in Hong Kong.

## Introduction

Myopia is the most common ocular disorder worldwide.^1,2^ The risk of ocular pathological changes increases with myopia due to excessive axial elongation, resulting in sight-threatening complications such as myopia maculopathy, retinal detachment, choroidal neovascularization, cataracts, and glaucoma.^3,4^ In particular, children with early onset of myopia have a higher risk of developing high myopia due to the longer duration of the disease and its faster progression.^5,6^ Childhood myopia is a significant public health concern, especially in East Asia.^7,8,9^ Recently, the outbreak of COVID-19 has worsened the social burdens of myopia owing to home confinement and the consequent lifestyle changes.^10,11^ A previous investigation^11^ found that the estimated annual incidence of myopia increased significantly from 11.6% in the pre–COVID-19 cohort to 29.7% in the COVID-19 cohort, indicating a potential myopia boom.

Both genetic and environmental factors contribute to the development of myopia.^12,13^ Besides established environmental factors such as increased near-work time and decreased outdoor time,^7,14,15^ exposure to nicotine, the most abundant chemical compound in cigarette smoke,^16^ has been revealed to be a potential risk factor. In experiments with chicks, nicotine has been found to induce myopia shifts,^17^ whereas nicotinic antagonists against neural nicotinic acetylcholine receptors inhibited myopia.^18^ In children, nicotine exposure is significantly higher in those with secondhand smoke (SHS) exposure.^19^ Secondhand smoke also contains hundreds of other chemicals apart from nicotine that are detrimental to health. In 2019, SHS was reportedly linked to 50 000 deaths with 4 500 000 disability-adjusted life-years among children (aged <14 years) worldwide.^20,21^ Globally, up to 40% of children are exposed to SHS^22^ and live with its pathophysiological disturbances to multiple organs, including the eyes.

In children, SHS exposure leads to higher risks of asthma, ear infections, respiratory tract infections, sudden infant death syndrome, and even biological changes in DNA.^23,24,25,26^ Notably, investigations by Yuan et al^27^ and Li et al^28^ revealed the structural effects on the choroid and retinal nerve fiber layer that can cause damage in children’s eyes and vision due to SHS exposure. Previous epidemiological studies have reported varied associations between SHS exposure and myopia. For instance, a prospective birth cohort study^29^ found that SHS exposure within 6 months after birth significantly increased the risk of developing myopia among 3-year-old children. In contrast, some investigations^30,31^ have reported that SHS exposure was associated with shifts toward hyperopia. If proven to be a health hazard to children, SHS exposure can affect the design, promotion, and implementation of public health policies. These conflicting findings warrant further exploration. This study seeks to address the research gap by examining the association between SHS exposure and myopia. Based on the potential biological pathway and evidence from the birth cohort study, we hypothesized that SHS exposure could be positively associated with myopia.

## Methods

### Study Population

Participants for this cross-sectional investigation were recruited from the Hong Kong Children Eye Study, a population-based study among schoolchildren aged 6 to 8 years in Hong Kong, China.^9^ Data were collected from March 15, 2015, to September 12, 2021. All children participated in comprehensive ophthalmic and physical examinations. Exclusion criteria included prior eye trauma, congenital malformations, ocular diseases (except myopia and hypermetropia), history of ocular surgery, and inability to complete ocular examinations. The study procedure was performed in accordance with the Declaration of Helsinki.^32^ The study protocol was approved by the Ethics Committee Board of The Chinese University of Hong Kong. Written informed consent was obtained from the legal guardians and oral assent was obtained from the children. This study followed the Strengthening the Reporting of Observational Studies in Epidemiology (STROBE) reporting guideline for cross-sectional studies.

### Refraction and Ocular Examinations

Trained ophthalmologists conducted complete ocular examinations for each participant, including examinations of the anterior segment, posterior segment, and ocular motility. Refraction was measured before and after cycloplegia using an autorefractor (ARK-510A; Nidek Incorporated). Two cycles of cycloplegic eye drops, including 1% cyclopentolate (Cyclogyl; Alcon-Convreur) and 1% tropicamide (Santen Pharmaceutical), were administered 10 minutes apart to perform cycloplegia. If a pupillary light reflex was still present or pupil size remained less than 6.0 mm, a third cycle of eye drops was administered 30 minutes later, and further cycles were administered as necessary to ensure sufficient pupil dilation. Ocular axial length (AL) was measured using an optical biometer (Zeiss IOLMaster; Carl Zeiss Meditec Inc).

### Definitions

Spherical equivalent (SE) was defined as spherical diopters (D) plus one-half of cylindrical D. Myopia was defined as an SE value of −0.50 D or less.^9^ Mild myopia was defined as SE of −3.00 D to −0.50 D; moderate myopia, as SE of −6.00 to −3.00 D; and high myopia, as SE of −6.00 D or less.^9^

### Questionnaires

During onsite interviews, the parents or legal guardians of each participating child completed validated questionnaires with assistance from trained staff. Missing and uncertain information was confirmed through additional telephone calls. Smoking behaviors of parents and other family members living with the children were examined to determine children’s SHS exposure collectively.^27,28^ The nonexposure group comprised children whose family members did not smoke or only smoked away from their home, and the exposure group comprised children living with 1 or more smokers in their family. The extent of SHS exposure was quantified as the daily total of cigarettes smoked at home by all smokers in the family.^27,28^ Information about SHS exposure was obtained through the following questions: (1) Has the mother smoked at home after the child was born? How long has she been smoking, and how many cigarettes a day? (2) Has the father smoked at home after the child was born? How long has he been smoking, and how many cigarettes a day? (3) Have other family members smoked at home after the child was born? How long have they each been smoking, and how many cigarettes a day?

The questionnaires also recorded information about the children’s age at myopia onset, daily living routine, living environment, lifestyle, and daily time spent outdoors (for exercise and leisure) and near work. To account for accommodative efforts and task proximity, near-work time was calculated in diopter-hours using the formula of 3 times (hours spent studying plus reading plus using cellphones) plus 2 times (hours spent on video games plus computer work) plus television hours.^33,34^

### Physical Examinations

Blood pressure was measured using a digital automatic blood pressure monitor (Spacelabs Healthcare). Height and weight were measured using an integrated professional device (Seca). Body mass index (BMI) was calculated as body weight in kilograms divided by height in meters squared.

### Statistical Analysis

The demographic characteristics of participants were summarized using descriptive statistics. Continuous variables are reported as means (SDs), while categorical variables are reported as numbers and percentages. Group differences in data were tested using an unpaired *t* test for continuous variables and a Pearson χ^2^ test for categorical variables. Two models were constructed for statistical analysis. Model 1 included the established fundamental confounders of age, sex, and parental myopia.^9,35^ Model 2 added other well-recognized confounders of BMI, outdoor time, near-work time, and family income,^36,37,38,39^ and the interaction effects of age and SHS exposure on myopia were further tested. Analyses of the association between SHS exposure and ocular parameters (SE and AL) were eye-based; data from both eyes were included, using generalized estimating equations with standard errors to adjust for intereye correlation.

Logistic regression was used to calculate the association between SHS exposure and myopia severity in a participant-based analysis. Moreover, linear regression was used to estimate the association between SHS exposure and age at myopia onset; while model 1 was not adjusted for any confounders, model 2 was adjusted for the genetic and fetal risk factors of sex, birth weight, and parental myopia.^40,41^ All statistical analyses were performed using SPSS, version 24.0 (IBM Corporation); 2-sided *P* < .05 was considered statistically significant. Subject to missing data, we used complete-case analyses without imputation.

## Results

### Study Sample

A total of 12 630 children aged 6 to 8 years (mean [SD] age, 7.37 [0.88] years; 5913 girls [46.8%] and 6717 boys [53.2%]) completed the ocular and physical examinations, in addition to the questionnaires. Among these, 444 participants (3.5%) had missing data. Based on conditions of smoking in the family, there were 4092 children (32.4%) in the SHS exposure group and 8538 children (67.6%) in the nonexposure group. The 2 groups were similar in age, sex, and time spent outdoors. However, children in the exposure group had greater BMI, birth weight, and near-work time; a lower prevalence of parental myopia; and lower family income and parental educational levels (Table 1). Household members smoked a mean (SD) of 10.5 (5.8) cigarettes daily.

**Table 1.** Demographic Characteristics of Study Individuals With and Without SHS Exposure (N = 12 630)^a^

Characteristic	No SHS Exposure (n = 8538)	SHS Exposure (n = 4092)	*P* value^b^
Age, mean (SD), y	7.37 (0.88)	7.36 (0.86)	.61
Sex			
Boys	4551 (53.3)	1263 (52.9)	.64
Girls	3987 (46.7)	1929 (47.1)	
Ratio of boys to girls	1.14	1.12	.61
BMI, mean (SD)	15.85 (2.39)	16.17 (2.49)	<.001
Birth weight, mean (SD), kg	2.99 (0.70)	3.08 (0.63)	<.001
Outdoor time, mean (SD), h/d^c^	1.09 (0.84)	1.09 (0.85)	.97
Near-work, diopter-hours/d^d^	9.11 (4.49)	9.46 (4.65)	<.001
Parental myopia^e^			
Yes	6569 (76.9)	2674 (65.3)	<.001
No	1969 (23.1)	1418 (34.7)	
Family income per month, HK $^f^			
≥20 000	6363 (74.5)	2337 (57.1)	<.001
<20 000	2175 (25.5)	1755 (42.9)	
Parents’ educational level			
Mother			
High school	4725 (55.3)	3304 (80.7)	<.001
Bachelor’s degree or higher	3813 (44.7)	788 (19.3)	
Father			
High school	4356 (51.0)	3341 (81.6)	<.001
Bachelor’s degree or higher	4182 (49.0)	751 (18.4)	

Abbreviations: BMI, body mass index (calculated as weight in kilograms divided by height in meters squared); SHS, secondhand smoke.

^a^ Unless otherwise specified, data are expressed as No. (%) of participants.

^b^ Statistical significance was tested using an independent unpaired *t* test; χ^2^ test was used to test the group difference in categorical data.

^c^ Outdoor time indicates outdoor exercise time plus outdoor leisure time.

^d^ Calculated using the following: [3 × hours spent studying + reading + using cellphones] + [2 × hours spent on video games plus computer work] + television hours.

^e^ At least 1 parent has myopia.

^f^ Equivalent of US $2551.10.

### Associations of SHS Exposure With SE and AL

Table 2 presents the associations of SHS exposure with SE and AL based on the generalized estimating equation analysis. After adjusting for age, sex, parental myopia, BMI, outdoor time, near-work time, and family income, model 2 showed that SHS exposure was associated with a 0.09-D SE decrease (β = −0.09 [95% CI, −0.14 to −0.03]) and a 0.05-mm AL increase (β = 0.05 [95% CI, 0.02-0.08]). Moreover, exposure to 10 cigarettes per day was associated with a 0.07-D SE decrease (β = −0.07 [95% CI, −0.11 to −0.02]) and a 0.04-mm AL increase (β = 0.04 [95% CI, 0.01-0.06]) (Table 3).

**Table 2.** Association of SHS Exposure With Spherical Equivalent and Axial Length in Children Aged 6 to 8 Years^a^

	Model 1		Model 2	
	β Coefficient (95% CI)	*P* value	β Coefficient (95% CI)	*P* value
**Spherical equivalent, D**				
SHS exposure (reference category is none)	−0.09 (−0.15 to −0.04)	.001	−0.09 (−0.14 to −0.03)	.003
Age, y	−0.41 (−0.44 to −0.38)	<.001	−0.42 (−0.45 to −0.39)	<.001
Sex (reference category is male)	0.10 (0.05 to 0.15)	<.001	0.11 (0.06 to 0.16)	<.001
No. of parents with myopia	−0.35 (−0.38 to −0.31)	<.001	−0.36 (−0.39 to −0.32)	<.001
BMI	NA	NA	0.003 (−0.01 to 0.01)	.55
Outdoor time, h/d^b^	NA	NA	0.04 (0.01 to 0.07)	.003
Near-work time, diopter-hours per day^c^	NA	NA	0.002 (−0.004 to 0.01)	.47
Family income (reference category <HK $20 000)^d^	NA	NA	0.07 (0.02 to 0.13)	.01
**Axial length, mm**				
SHS exposure (reference category is none)	0.06 (0.02 to 0.09)	.001	0.05 (0.02 to 0.08)	.003
Age, y	0.33 (0.32 to 0.35)	<.001	0.32 (0.31 to 0.34)	<.001
Sex (reference category is male)	−0.55 (−0.58 to −0.52)	<.001	−0.54 (−0.57 to −0.51)	<.001
No. of parents with myopia	0.14 (0.12 to 0.15)	<.001	0.14 (0.12 to 0.16)	<.001
BMI	NA	NA	0.02 (0.01 to 0.03)	<.001
Outdoor time, h/d^b^	NA	NA	−0.01 (−0.03 to 0.01)	.15
Near-work time, diopter-hours per day^c^	NA	NA	−0.002 (−0.01 to 0.001)	.23
Family income (reference category <HK $20 000)^d^	NA	NA	−0.02 (−0.05 to 0.01)	.26

Abbreviations: BMI, body mass index (calculated as weight in kilograms divided by height in meters squared); D, diopter; NA, not applicable; SHS, secondhand smoke.

^a^ Generalized estimating equations were used to adjust the correlation between eyes.

^b^ Indicates outdoor exercise time plus outdoor leisure time.

^c^ Calculated using the following: [3 × hours spent studying + reading + using cellphones] + [2 × hours spent on video games plus computer work] + television hours.

^d^ Equivalent of US $2551.10.

**Table 3.** Association of Quantity of SHS Exposure With Spherical Equivalent and Axial Length in Children Aged 6 to 8 Years^a^

	Model 1		Model 2	
	β Coefficient (95% CI)	*P* value	β Coefficient (95% CI)	*P* value
**Spherical equivalent, D**				
Smoking quantity, 10 cigarettes per 1 U	−0.07 (−0.12 to −0.03)	.001	−0.07 (−0.11 to −0.02)	.004
Age, y	−0.41 (−0.44 to −0.38)	<.001	−0.42 (−0.45 to −0.39)	<.001
Sex (reference category is male)	0.10 (0.05 to 0.15)	<.001	0.11 (0.06 to 0.16)	<.001
No. of parents with myopia	−0.34 (−0.38 to −0.31)	<.001	−0.36 (−0.39 to −0.32)	<.001
BMI	NA	NA	0.003 (−0.01 to 0.01)	.56
Outdoor time, h/d^b^	NA	NA	0.04 (0.01 to 0.07)	.004
Near-work time, diopter-hours per day^c^	NA	NA	0.002 (−0.004 to 0.01)	.50
Family income (reference category <HK $20 000)^d^	NA	NA	0.07 (0.02 to 0.13)	.01
**Axial length, mm**				
Smoking quantity, 10 cigarettes per 1 U	0.04 (0.02 to 0.07)	.001	0.04 (0.01 to 0.06)	.005
Age, y	0.33 (0.32 to 0.35)	<.001	0.32 (0.31 to 0.34)	<.001
Sex (reference category is male)	−0.55 (−0.58 to −0.52)	<.001	−0.54 (−0.57 to −0.51)	<.001
No. of parents with myopia	0.13 (0.12 to 0.15)	<.001	0.14 (0.12 to 0.16)	<.001
BMI	NA	NA	0.02 (0.01 to 0.03)	<.001
Outdoor time, h/d^b^	NA	NA	−0.01 (−0.03 to 0.01)	.16
Near-work time, diopter-hours per day^c^	NA	NA	−0.002 (−0.01 to 0.001)	.24
Family income (reference category <HK $20 000)^d^	NA	NA	−0.02 (−0.05 to 0.01)	.24

Abbreviations: BMI, body mass index (calculated as weight in kilograms divided by height in meters squared); D, diopter; NA, not applicable; SHS, secondhand smoke.

^a^ Generalized estimating equations were used to adjust the correlation between eyes.

^b^ Indicates outdoor exercise time plus outdoor leisure time.

^c^ Calculated using the following: [3 × hours spent studying + reading + using cellphones] + [2 × hours spent on video games plus computer work] + television hours.

^d^ Equivalent of US $2551.10.

There were significant interactions between age and SHS exposure on SE and AL (eTable 1 in Supplement 1 presents values with increasing age), showing that the association between SHS exposure and SE and AL was magnified in younger children. For each year younger of a child’s exposure to SHS, SHS exposure was associated with a 0.07-D decrease in SE (β = 0.07 [95% CI, 0.01-0.13]) and a 0.05-mm increase in AL (β = −0.05 [95% CI, −0.08 to −0.01]); exposure to 10 cigarettes per day was associated with a 0.06-D SE decrease (β = 0.06 [95% CI, 0.01-0.11]) and a 0.04-mm AL increase (β = −0.04 [95% CI, −0.07 to −0.01]). The subgroup analysis further illustrated more significant associations among younger children (eTable 2 in Supplement 1).

### Association of SHS Exposure With Myopia

In model 2, children with SHS exposure were 1.30 (95% CI, 1.06-1.59) and 2.64 (95% CI, 1.48-4.69) times more likely to develop moderate and high myopia, respectively, compared with the nonexposure group (Table 4). The greater the daily SHS exposure, the higher the odds of developing moderate (odds ratio [OR], 1.23 [95% CI, 1.05-1.44]) and high (OR, 1.75 [95% CI, 1.20-2.56]) myopia.

**Table 4.** Association of SHS Exposure and Odds of Different Severities of Myopia in Children Aged 6 to 8 Years^a^

	Any myopia		Mild myopia		Moderate myopia		High myopia	
	OR (95% CI)	*P* value	OR (95% CI)	*P* value	OR (95% CI)	*P* value	OR (95% CI)	*P* value
**SHS exposure in children (reference category is none)**								
Model 1^b^	1.08 (0.99-1.18)	.10	1.03 (0.94-1.13)	.57	1.28 (1.05-1.56)	.02	2.74 (1.56-4.82)	<.001
Model 2^c^	1.07 (0.98-1.17)	.14	1.02 (0.92-1.12)	.73	1.30 (1.06-1.59)	.01	2.64 (1.48-4.69)	.001
**Smoking quantity, 10 cigarettes as 1 unit**								
Model 1^b^	1.08 (1.00-1.15)	.04	1.04 (0.96-1.12)	33	1.22 (1.05-1.43)	.01	1.81 (1.25-2.63)	.002
Model 2^c^	1.07 (1.00-1.15)	.07	1.03 (0.95-1.11)	.23	1.23 (1.05-1.44)	.01	1.75 (1.20-2.56)	.004

Abbreviations: OR, odds ratio; SHS, secondhand smoke.

^a^ Smoking pack-years indicates number of cigarettes/20 × smoking-years after the child was born. Interaction of age and SHS (results of model 3) was not significant for myopia prevalence. Children without myopia were the reference group. *P* values were generated by logistic regression.

^b^ Adjusted for age, sex, and parental myopia.

^c^ Adjusted for age, sex, parental myopia, body mass index, near-work time, outdoor time, and family income.

### Association of SHS Exposure With Age at Myopia Onset

There were negative associations between SHS exposure and the age at myopia onset (Table 5). After adjusting for sex, birth weight, and parental myopia, SHS exposure was associated with an earlier mean (SD) age at myopia onset compared with children who were not exposed to SHS (72.8 [0.9] vs 74.6 [0.6] months; *P* = .01). Moreover, every 10 more cigarettes smoked in the family per day was associated with a 1-month advance in myopia onset (β = −1.30 [95% CI, −2.32 to −0.27]).

**Table 5.** Association of SHS Exposure With Self-reported Age at Myopia Onset in Children^a^

	Self-reported age of myopia onset, mo			
	Model 1^b^		Model 2^c^	
	β Coefficient (95% CI)	*P* value	β Coefficient (95% CI)	*P* value
SHS exposure in children, no as reference	−1.57 (−2.91 to −0.23)	.02	−1.80 (−3.16 to −0.43)	.01
Smoking quantity, 10 cigarettes per 1 U	−1.12 (−2.12 to −0.11)	.03	−1.30 (−2.32 to −0.27)	.01

Abbreviation: SHS, secondhand smoke.

^a^ Linear regression was used to calculate the *P* values.

^b^ Indicates without adjustment.

^c^ Adjusted for sex, birth weight, and parental myopia.

## Discussion

In line with previous local studies examining household SHS exposure among children (31.5%),^42^ adolescents (33.2%)^43^ and adults (33.1%),^44^ the present study found that about 1 in 3 children in Hong Kong had SHS exposure at home. Exposure to SHS presents a dose-dependent association with myopia among children aged 6 to 8 years. Exposure to SHS was associated with greater myopic refraction, longer AL, higher odds of developing more severe myopia, and earlier age at myopia onset. Moreover, greater SHS exposure was associated with higher myopic shifts and earlier myopia onset. The younger the children, the more significant the association between SHS exposure and myopia.

After adjusting for potential confounders, SHS exposure was associated with a 0.09-D SE decrease and 0.05-mm AL elongation. Demographically similar studies reported annual myopia progressions ranging from −0.27 to −0.42 D in refraction and 0.27 mm in AL.^45,46,47^ Using these data as references, the SHS-associated differences in our study account for approximately one-fifth (0.09/0.42 and 0.05/0.27) to one-third (0.09/0.27) of the progression in a year, equaling about 2- to 4-month progression. Similarly, daily exposure to 10 cigarettes was associated with a 0.07-D SE decrease and a 0.04-mm AL increase, which was about 2 to 3 months of progression.

A similar association between SHS exposure and myopia had been reported for the Growing Up in Singapore Toward Healthy Outcomes (GUSTO) birth cohort.^29^ Among 572 children aged 3 years, SHS exposure within 6 months after birth was associated with an increased risk of myopia (OR, 2.79 [95% CI, 1.24-6.29]; *P* = .01). However, possibly due to the low prevalence rate for myopia in this age group, the GUSTO study did not find any associations between SHS exposure and SE and AL. Conversely, our study focused on schoolchildren and demonstrated for the first time the association between SHS exposure and mean values of SE and AL. Furthermore, our study found that SHS exposure was associated with more severe myopia among schoolchildren. In the GUSTO study, children were more likely to develop early-onset myopia with SHS exposure, which might progress further to higher severity later in life with continuous SHS exposure, echoing findings of our study.

Contrary to our findings, a negative association between SHS exposure and myopia was frequently reported. In Egypt, 300 children aged 5 to 12 years with hyperopia had higher urinary cotinine levels, which is the end product of nicotine metabolism, than children with myopia or emmetropia.^30^ Among 323 individuals aged 1 to 20 years in the US, participants with at least 1 parent who smoked had lower myopia prevalence (12.4% vs 25.4%; *P* = .004) and exhibited greater mean (SD) hyperopic refractions (1.83 [0.24] vs 0.96 [0.27] D; *P* = .02).^31^ Since 25.4% of the study participants were aged 12 to 20 years, some of them may have been tobacco smokers.^48^

Younger children may be potentially exposed to more SHS chemical components, as they have faster respiratory rates and breathe more air relative to their body mass.^49,50^ Moreover, younger children have limited control over their living environment and less awareness of the risks of SHS for self-protection.^51^ Regarding an SHS-related myopic shift, a possible mechanism is the activation of nicotinic acetylcholine receptors localized at the retina and other ocular tissues by nicotine,^52,53,54^ which may have dose-dependent effects. Furthermore, younger children with a less developed visual system are more sensitive to conditions, such as chemical components in SHS, that interfere with visual development.^55,56^ Last, since times of near work and outdoor activities play stronger roles in myopia development and progression in more senior school years, the role of SHS exposure may become marginal, weakening the association between SHS exposure and myopia in older children.^57,58^ Our findings suggest that earlier SHS avoidance is of greater importance in myopia management in schoolchildren.

Our results showed the association of SHS exposure with earlier myopia onset, a risk factor for high myopia later in life. A 12-year longitudinal study^59^ found that each year of delay in the age of myopia onset could drastically decrease the possibility of high myopia in adulthood (OR, 0.44 [95% CI, 0.36-0.55]; *P* < .001). Our study showed that children with SHS exposure exhibited an onset of myopia approximately 2 months earlier than children without exposure, which echoes our speculated clinical significance of accumulated differences in SE and AL as discussed earlier.

### Limitations

This study has some limitations. Hong Kong has strictly prohibited public smoking since 2007, where SHS exposure from family members accurately reflects children’s SHS exposure.^60^ We thereby considered SHS exposure status a binary variable without specifying the family members who smoked. Specifically, paternal smoking contributed dominantly to SHS exposure (≥80%), revealing consistent results with the main results (eTable 3 in Supplement 1). Due to the limited rate (around 5%) and power of maternal smoking, we did not test its association with myopia. Regarding the extent of smoking exposure, we focused on current smoking quantity but not duration, as retrospective smoking history may not be reliable over a long period.^61^ However, using pack-years by multiplying the daily cigarettes’ packs by the years a child has been exposed, similar results emerged that more pack-years were associated with greater myopic refraction and AL elongation (eTable 4 in Supplement 1).

Other limitations include that we did not regard children with family members smoking outside as having SHS exposure. Family members might have underreported their smoking at home, although most smokers living with children tend to report that smoking occurs in their home every day,^62^ and our study reported a rate of SHS exposure consistent with that of previous studies.^42,43,44^ Including children whose family members only smoked away from home in the nonexposure group might have reduced the between-group differences and underestimated the association between SHS exposure and myopia.

The interaction between age and the association of SHS exposure and myopia was only revealed in a narrow range. Exposure to SHS possibly began since birth or during pregnancy, and 6 to 8 years only accounts for a short period in lifetime exposure. However, as physical, social, and mental conditions develop quickly during middle childhood (6-8 years of age),^63^ children are susceptible to environmental factors,^64^ and their responses may vary substantially during this short period.^65^ Myopia develops particularly fast, showing the most drastic increase in prevalence from 6 (14.8%) to 7 (38.5%) years of age among Chinese children.^66^

Due to their high correlations, family income, but not parental educational level, was adjusted as a socioeconomic indicator. Of note, consistent results were generated when including parental educational level in the models (eTable 5 in Supplement 1). To tease out any residual confounding from family income, we further evaluated the models among groups stratified by family income (eTable 6 in Supplement 1), showing children in high-income families were particularly vulnerable to SHS exposure regarding myopia. The role of family income in the association between SHS exposure and myopia remains to be explored. Also, besides the well-recognized confounders, other myopia-related contextual factors (such as school operating systems) might vary from society to society.^67^ Future studies are warranted to confirm the association in other populations.

Furthermore, our study was limited by its cross-sectional design, which cannot conclude a causative relationship, warranting further longitudinal evidence. Nevertheless, SHS exposure status and quantity accuracy could be subject to recall and reporting biases.

## Conclusions

The findings of this cross-sectional study suggest that SHS exposure was associated with greater mean myopic refraction, longer AL, higher odds of moderate and high myopia, and earlier onset of myopia. The higher the SHS exposure and the younger the child, the more advanced myopia development and progression with which SHS exposure is associated. Eliminating SHS exposure for eye care among children is important, particularly in families with young children.

## Notes

Supplement 1. **eTable 1.** Interaction Effect of Secondhand Smoke (SHS) Exposure and Age on Spherical Equivalent and Axial Length in Children Aged 6 to 8 Years **eTable 2.** Association of Secondhand Smoke (SHS) Exposure With Spherical Equivalent and Axial Length Among Different Aged Children **eTable 3.** Association of Quantity of Paternal Secondhand Smoke (SHS) Exposure With Spherical Equivalent and Axial Length in Children Aged 6 to 8 Years **eTable 4.** Association of Smoking Pack-years With Spherical Equivalent and Axial Length in Children Aged 6 to 8 Years **eTable 5.** Association of Secondhand Smoke (SHS) Exposure With Spherical Equivalent and Axial Length in Children Aged 6 to 8 Years (With Parental Education Levels Included) **eTable 6.** Association of Secondhand Smoke (SHS) Exposure With Spherical Equivalent and Axial Length Stratified by Family Income

Supplement 2.
Data Sharing Statement
