# Supplementary material for: Analysis of Secondhand Smoke Exposure and Myopia Among Children Aged 6 to 8 Years in Hong Kong
Source: JAMA Netw Open. 2023 May 11;6(5):e2313006. doi: 10.1001/jamanetworkopen.2023.13006 (PMC10176122; doi:10.1001/jamanetworkopen.2023.13006)
Supplement: Supplement 1. — eTable 1. Interaction Effect of Secondhand Smoke (SHS) Exposure and Age on Spherical Equivalent and Axial Length in Children Aged 6 to 8 Years eTable 2. Association of Secondhand Smoke (SHS) Exposure With Spherical Equivalent and Axial Length Among Different Aged Children eTable 3. Association of Quantity of Paternal Secondhand Smoke (SHS) Exposure With Spherical Equivalent and Axial Length in Children Aged 6 to 8 Years eTable 4. Association of Smoking Pack-years With Spherical Equivalent and Axial Length in Children Aged 6 to 8 Years eTable 5. Association of Secondhand Smoke (SHS) Exposure With Spherical Equivalent and Axial Length in Children Aged 6 to 8 Years (With Parental Education Levels Included) eTable 6. Association of Secondhand Smoke (SHS) Exposure With Spherical Equivalent and Axial Length Stratified by Family Income [file jamanetwopen-e2313006-s001.pdf]

Supplementary Online Content

Zhang Y, Zhang XJ, Yuan N, et al. Analysis of secondhand smoke exposure and myopia among children aged 6 to 8 years in Hong Kong. *JAMA Netw Open*. 2023;6(5):e2313006. doi:10.1001/jamanetworkopen.2023.13006

eTable 1. Interaction Effect of Secondhand Smoke (SHS) Exposure and Age on Spherical Equivalent and Axial Length in Children Aged 6 to 8 Years
eTable 2. Association of Secondhand Smoke (SHS) Exposure With Spherical Equivalent and Axial Length Among Different Aged Children
eTable 3. Association of Quantity of Paternal Secondhand Smoke (SHS) Exposure With Spherical Equivalent and Axial Length in Children Aged 6 to 8 Years
eTable 4. Association of Smoking Pack-years With Spherical Equivalent and Axial Length in Children Aged 6 to 8 Years
eTable 5. Association of Secondhand Smoke (SHS) Exposure With Spherical Equivalent and Axial Length in Children Aged 6 to 8 Years (With Parental Education Levels Included)
eTable 6. Association of Secondhand Smoke (SHS) Exposure With Spherical Equivalent and Axial Length Stratified by Family Income

This supplementary material has been provided by the authors to give readers additional information about their work.

© 2023 Zhang Y et al. *JAMA Network Open*.

eTable 1. Interaction Effect of Secondhand Smoke (SHS) Exposure and Age on Spherical Equivalent and Axial Length in Children Aged 6 to 8 Years

	**Spherical equivalent, D**		**Axial length, mm**	
	β (95%CI)	*P* value	β (95%CI)	*P* value
**SHS exposure**				
SHS exposure, no as reference	-0.58 (-1.04 to -0.13)	0.01	0.41 (0.15 to 0.67)	0.002
Age, years	-0.44 (-0.47 to -0.40)	<0.001	0.34 (0.32 to 0.36)	<0.001
Sex, male as reference	0.11 (0.06 to 0.16)	<0.001	-0.54 (-0.57 to -0.51)	<0.001
No. of myopic parents	-0.36 (-0.39 to -0.32)	<0.001	0.14 (0.12 to 0.16)	<0.001
BMI, kg/m^2	0.003 (-0.01 to 0.01)	0.54	0.02 (0.01 to 0.03)	<0.001
Outdoor time, hours per day [a]	0.04 (0.01 to 0.07)	0.004	-0.01 (-0.03 to 0.01)	0.15
Near work, diopter hours per day [b]	0.002 (-0.004 to 0.01)	0.48	-0.002 (-0.01 to 0.001)	0.23
Family income, <HK $20000 [c] as reference	0.07 (0.02 to 0.13)	0.01	-0.02 (-0.05 to 0.01)	0.27
Age*SHS exposure	0.07 (0.01 to 0.13)	0.03	-0.05 (-0.08 to -0.01)	0.008
**SHS quantity**				
Smoking quantity, 10 cigarettes as 1 unit	-0.50 (-0.87 to -0.12)	0.009	0.32 (0.12 to 0.53)	0.002
Age, years	-0.44 (-0.47 to -0.40)	<0.001	0.34 (0.32 to 0.36)	<0.001
Sex, male as reference	0.11 (0.06 to 0.16)	<0.001	-0.54 (-0.57 to -0.51)	<0.001
No. of myopic parents	-0.36 (-0.39 to -0.32)	<0.001	0.14 (0.12 to 0.16)	<0.001
BMI, kg/m^2	0.003 (-0.01 to 0.02)	0.56	0.02 (0.01 to 0.03)	<0.001
Outdoor time, hours per day [a]	0.04 (0.01 to 0.07)	0.004	-0.01 (-0.03 to 0.01)	0.16
Near work, diopter hours per day [b]	0.002 (-0.004 to 0.01)	0.49	-0.002 (-0.01 to 0.001)	0.24
Family income, <HK $20000 [c] as reference	0.07 (0.02 to 0.13)	0.01	-0.02 (-0.05 to 0.01)	0.24
Age*Smoking quantity	0.06 (0.01 to 0.11)	0.03	-0.04 (-0.07 to -0.01)	0.007
**SHS pack-years**				
Smoking pack-years, 1 pack/day for 1 year as 1 unit [c]	-0.16 (-0.27 to -0.05)	0.006	0.10 (0.04 to 0.16)	0.002
Age, years	-0.43 (-0.47 to -0.40)	<0.001	0.34 (0.32 to 0.36)	<0.001
Sex, male as reference	0.11 (0.06 to 0.16)	<0.001	-0.54 (-0.57 to -0.51)	<0.001
No. of myopic parents	-0.36 (-0.39 to -0.32)	<0.001	0.14 (0.12 to 0.16)	<0.001
BMI, kg/m^2	0.003 (-0.01 to 0.01)	0.56	0.02 (0.01 to 0.03)	<0.001
Outdoor time, hours per day [a]	0.04 (0.01 to 0.72)	0.004	-0.01 (-0.03 to 0.01)	0.16
Near work, diopter hours per day [b]	0.002 (-0.004 to 0.01)	0.48	-0.002 (-0.01 to 0.001)	0.24
Family income, <HK $20000 [c] as reference	0.07 (0.02 to 0.13)	0.01	-0.02 (-0.05 to 0.01)	0.23
Age*Smoking quantity	0.02 (0.004 to 0.03)	0.02	-0.01 (-0.02 to -0.004)	0.005

© 2023 Zhang Y et al. *JAMA Network Open*.

D = diopter, β = beta coefficient, SE = standard error.
Generalized estimating equations (GEEs) were used to adjust the correlation between eyes.
[a]. Outdoor time = outdoor exercise time + outdoor leisure time.
[b]. Near work = 3*(homework + reading + playing cell phone) + 2*(using computer + playing video game) +1*(watching TV).
[c]. US $2551.10.
[d]. Smoking pack-years = cigarette number/20* smoking years after the child was born.

© 2023 Zhang Y et al. *JAMA Network Open*.

eTable 2. Association of Secondhand Smoke (SHS) Exposure With Spherical Equivalent and Axial Length Among Different Aged Children

**Age (years)**	**Spherical Equivalent, D, mean (95%CI)**				**Axial Length, mm, mean (95%CI)**			
	With SHS exposure	Without SHS exposure	Estimated mean difference (95%CI)	*P* value	With SHS exposure	Without SHS exposure	Estimated mean difference (95%CI)	*P* value
6.00-6.49 (n=2314)	0.62 (0.54, 0.70)	0.73 (0.67, 0.79)	-0.11 (-0.21, -0.01)	0.03	22.69 (22.64, 22.74)	22.61 (22.58, 22.65)	0.08 (0.01, 0.14)	0.02
6.50-6.99 (n=2337)	0.29 (0.17, 0.42)	0.47 (0.38, 0.55)	-0.17 (-0.33, -0.02)	0.03	22.93 (22.88, 22.98)	22.86 (22.82, 22.90)	0.07 (0.003, 0.13)	0.04
7.00-7.49 (n=2407)	0.19 (0.09, 0.29)	0.32 (0.25, 0.39)	-0.12 (-0.24, -0.002)	0.04	23.08 (23.01, 23.15)	23.00 (22.95, 23.04)	0.08 (0.001, 0.16)	0.04
7.50-7.99 (n=2125)	0.03 (-0.09, 0.14)	0.09 (0.01, 0.17)	-0.06 (-0.20, 0.07)	0.37	23.21 (23.14, 23.27)	23.17 (23.13, 23.22)	0.04 (-0.04, 0.11)	0.38
8.00-8.49 (n=1871)	-0.18 (-0.31, -0.05)	-0.19 (-0.28, -0.09)	0.01 (-0.16, 0.17)	0.95	23.36 (23.28, 23.43)	23.35 (23.29, 23.40)	0.01 (-0.08, 0.10)	0.77
8.50-8.99 (n=1576)	-0.40 (-0.56, -0.25)	-0.36 (-0.47, -0.25)	-0.04 (-0.23, 0.14)	0.64	23.46 (23.38, 23.54)	23.45 (23.39, 23.51)	0.01 (-0.09, 0.10)	0.89

D = diopter, CI = confidence interval;
Estimated mean values were generated by generalized estimating equations (GEEs) with the adjustment of sex, parental myopia, BMI, near work, outdoor time, and family income.

© 2023 Zhang Y et al. *JAMA Network Open*.

eTable 3. Association of Quantity of Paternal Secondhand Smoke (SHS) Exposure With Spherical Equivalent and Axial Length in Children Aged 6 to 8 Years

	Model 1		Model 2	
	β (95%CI)	*P* value	β (95%CI)	*P* value
**Spherical Equivalent, D**				
Paternal smoking quantity, 10 cigarettes as 1 unit	-0.09 (-0.14 to -0.03)	0.001	-0.08 (-0.14 to -0.03)	0.004
Age, years	-0.43 (-0.46 to -0.40)	<0.001	-0.43 (-0.46 to -0.40)	<0.001
Sex, male as reference	0.10 (0.05 to 0.16)	<0.001	0.10 (0.05 to 0.16)	<0.001
No. of myopic parents	-0.34 (-0.37 to -0.30)	<0.001	-0.35 (-0.39 to -0.31)	<0.001
BMI, kg/m^2	NA	NA	0.001 (-0.01, 0.01)	0.83
Outdoor time, hours per day [a]	NA	NA	0.03 (0.00 to 0.07)	0.04
Near work, diopter hours per day [b]	NA	NA	0.003 (-0.004 to 0.01)	0.36
Family income, <HK $20000 [c] as reference	NA	NA	0.07 (0.01 to 0.14)	0.02
**Axial Length, mm**				
Paternal smoking quantity, 10 cigarettes as 1 unit	0.06 (0.02 to 0.09)	<0.001	0.05 (0.02 to 0.08)	0.002
Age, years	0.34 (0.32 to 0.36)	<0.001	0.33 (0.31 to 0.35)	<0.001
Sex, male as reference	-0.55 (-0.58 to -0.52)	<0.001	-0.54 (-0.57 to -0.51)	<0.001
No. of myopic parents	0.13 (0.11 to 0.15)	<0.001	0.14 (0.12 to 0.16)	<0.001
BMI, kg/m^2	NA	NA	0.02 (0.01 to 0.03)	<0.001
Outdoor time, hours per day [a]	NA	NA	-0.01 (-0.03 to 0.01)	0.18
Near work, diopter hours per day [b]	NA	NA	-0.001 (-0.01 to 0.002)	0.48
Family income, <HK $20000 [c] as reference	NA	NA	-0.02 (-0.06 to 0.02)	0.29

D = diopter, β = beta coefficient, CI = confidence interval.

Generalized estimating equations (GEEs) were used to adjust the correlation between eyes.

[a]. Outdoor time = outdoor exercise time + outdoor leisure time.

[b]. Near work = 3*(homework + reading + playing cell phone) + 2*(using computer + playing video game) +1*(watching TV).

[c]. US $2551.10.

© 2023 Zhang Y et al. *JAMA Network Open*.

eTable 4. Association of Smoking Pack-years With Spherical Equivalent and Axial Length in Children Aged 6 to 8 Years

	Model 1		Model 2	
	β (95%CI)	*P* value	β (95%CI)	*P* value
**Spherical Equivalent, D**				
Smoking Pack-years [a]	-0.02 (-0.03 to -0.01)	0.004	-0.02 (-0.03 to -0.004)	0.01
Age, years	-0.41 (-0.44 to -0.38)	<0.001	-0.41 (-0.44 to -0.38)	<0.001
Sex, male as reference	0.10 (0.05 to 0.15)	<0.001	0.11 (0.06 to 0.16)	<0.001
No. of myopic parents	-0.34 (-0.38 to -0.31)	<0.001	-0.35 (-0.39 to -0.32)	<0.001
BMI, kg/m^2	NA	NA	0.003 (-0.01 to 0.01)	0.57
Outdoor time, hours per day [b]	NA	NA	0.04 (0.01 to 0.07)	0.004
Near work, diopter hours per day [c]	NA	NA	0.002 (-0.004 to 0.01)	0.48
Family income, <HK $20000 [d] as reference	NA	NA	0.08 (0.02 to 0.13)	0.01
**Axial Length, mm**				
Smoking Pack-years [a]	0.01 (0.003 to 0.02)	0.007	0.01 (0.001 to 0.02)	0.02
Age, years	0.33 (0.32 to 0.35)	<0.001	0.32 (0.31 to 0.34)	<0.001
Sex, male as reference	-0.55 (-0.58 to -0.52)	<0.001	-0.54 (-0.57 to -0.51)	<0.001
No. of myopic parents	0.13 (0.11 to 0.15)	<0.001	0.14 (0.12 to 0.16)	<0.001
BMI, kg/m^2	NA	NA	0.02 (0.01 to 0.03)	<0.001
Outdoor time, hours per day [b]	NA	NA	-0.01 (-0.03 to 0.01)	0.15
Near work, diopter hours per day [c]	NA	NA	-0.002 (-0.01 to 0.001)	0.24
Family income, <HK $20000 [d] as reference	NA	NA	-0.02 (-0.05 to 0.01)	0.22

D = diopter, β = beta coefficient, CI = confidence interval.

Generalized estimating equations (GEEs) were used to adjust the correlation between eyes.

[a]. Smoking pack-years = cigarette number/20* smoking years after the child was born.

Outdoor time = outdoor exercise time + outdoor leisure time.

[b]. Near work = 3*(homework + reading + playing cell phone) + 2*(using computer + playing video game) +1*(watching TV).

[c]. US $2551.10.

© 2023 Zhang Y et al. *JAMA Network Open*.

eTable 5. Association of Secondhand Smoke (SHS) Exposure With Spherical Equivalent and Axial Length in Children Aged 6 to 8 Years (With Parental Education Levels Included)

	**Spherical Equivalent, D**		**Axial Length, mm**	
	β (95%CI)	*P* value	β (95%CI)	*P* value
**SHS exposure**				
SHS exposure, no as reference	-0.08 (-0.14, -0.02)	0.01	0.04 (0.01, 0.07)	0.02
Age, years	-0.42 (-0.44, -0.39)	<0.001	0.32 (0.31, 0.34)	<0.001
Sex, male as reference	0.11 (0.06, 0.16)	<0.001	-0.54 (-0.57, -0.51)	<0.001
No. of myopic parents	-0.38 (-0.42, -0.35)	<0.001	0.15 (0.13, 0.17)	<0.001
BMI, kg/m^2	0.004 (-0.01, 0.02)	0.44	0.02 (0.01, 0.03)	<0.001
Outdoor time, hours per day [a]	0.04 (0.01, 0.07)	0.006	-0.01 (-0.03, 0.01)	0.17
Near work, diopter hours per day [b]	0.002 (-0.004, 0.01)	0.45	-0.002 (-0.01, 0.001)	0.22
Maternal education level	0.08 (0.02, 0.15)	0.02	-0.03 (-0.06, 0.01)	0.18
Paternal education level	0.11 (0.05, 0.18)	<0.001	-0.03 (-0.06, 0.01)	0.18
**SHS quantity**				
Smoking quantity, 10 cigarettes as 1 unit	-0.06 (-0.11, -0.01)	0.02	0.03 (0.004, 0.06)	0.03
Age, years	-0.41 (-0.44, -0.39)	<0.001	0.32 (0.31, 0.34)	<0.001
Sex, male as reference	0.11 (0.06, 0.16)	<0.001	-0.54 (-0.57, -0.51)	<0.001
No. of myopic parents	-0.38 (-0.42, -0.35)	<0.001	0.15 (0.13, 0.17)	<0.001
BMI, kg/m^2	0.004 (-0.01, 0.02)	0.45	0.02 (0.01, 0.03)	<0.001
Outdoor time, hours per day [a]	0.04 (0.01, 0.07)	0.007	-0.01 (-0.03, 0.01)	0.18
Near work, diopter hours per day [b]	0.002 (-0.004, 0.01)	0.45	-0.002 (-0.01, 0.001)	0.23
Maternal education level	0.08 (0.02, 0.15)	0.02	-0.03 (-0.06, 0.01)	0.18
Paternal education level	0.12 (0.05, 0.18)	<0.001	-0.03 (-0.06, 0.01)	0.16

D = diopter, β = beta coefficient, SE = standard error.

Generalized estimating equations (GEEs) were used to adjust the correlation between eyes.

[a]. Outdoor time = outdoor exercise time + outdoor leisure time.

[b]. Near work = 3*(homework + reading + playing cell phone) + 2*(using computer + playing video game) +1*(watching TV).

[c]. US $2551.10.

© 2023 Zhang Y et al. *JAMA Network Open*.

eTable 6. Association of Secondhand Smoke (SHS) Exposure With Spherical Equivalent and Axial Length Stratified by Family Income

	**Spherical Equivalent, D**		**Axial Length, mm**	
	β (95%CI)	*P* value	β (95%CI)	*P* value
**Family income <20000 HKD [c]**				
SHS exposure, no as reference	-0.01 (-0.10, 0.08)	0.82	0.01 (-0.04, 0.06)	0.78
Age, years	-0.38 (-0.43, -0.32)	<0.001	0.28 (0.25, 0.32)	<0.001
Sex, male as reference	0.02 (-0.07, 0.10)	0.72	-0.51 (-0.56, -0.45)	<0.001
No. of myopic parents	-0.33 (-0.39, -0.27)	<0.001	0.14 (0.10, 0.17)	<0.001
BMI, kg/m^2	-0.0003 (-0.02, 0.02)	0.98	0.02 (0.01, 0.03)	<0.001
Outdoor time, hours per day [a]	0.07 (0.02, 0.12)	0.008	-0.02 (-0.05, 0.01)	0.10
Near work, diopter hours per day [b]	0.001 (-0.01, 0.01)	0.91	-0.002 (-0.01, 0.004)	0.45
**Family income ≥20,000 HKD [c]**				
SHS exposure, no as reference	-0.15 (-0.22, -0.08)	<0.001	0.07 (0.03, 0.11)	<0.001
Age, years	-0.43 (-0.47, -0.40)	<0.001	0.34 (0.32, 0.36)	<0.001
Sex, male as reference	0.15 (0.09, 0.21)	<0.001	-0.55 (-0.59, -0.52)	<0.001
No. of myopic parents	-0.37 (-0.42, -0.33)	<0.001	0.15 (0.12, 0.17)	<0.001
BMI, kg/m^2	0.01 (-0.01, 0.02)	0.43	0.02 (0.01, 0.03)	<0.001
Outdoor time, hours per day [a]	0.03 (-0.004, 0.07)	0.08	-0.01 (-0.03, 0.01)	0.48
Near work, diopter hours per day [b]	0.003 (-0.01, 0.01)	0.48	-0.002 (-0.01, 0.002)	0.39

D = diopter, β = beta coefficient, SE = standard error.

Generalized estimating equations (GEEs) were used to adjust the correlation between eyes.

[a]. Outdoor time = outdoor exercise time + outdoor leisure time.

[b]. Near work = 3*(homework + reading + playing cell phone) + 2*(using computer + playing video game) +1*(watching TV).

[c]. US $2551.10.

© 2023 Zhang Y et al. *JAMA Network Open*.
