# Supplementary material for: Analysis of Secondhand Smoke Exposure and Myopia Among Children Aged 6 to 8 Years in Hong Kong
Source: JAMA Netw Open. 2023 May 11;6(5):e2313006. doi: 10.1001/jamanetworkopen.2023.13006 (PMC10176122; doi:10.1001/jamanetworkopen.2023.13006)
Supplement: Supplement 2. — Data Sharing Statement [file jamanetwopen-e2313006-s002.pdf]

Data Sharing Statement

Zhang. Analysis of Secondhand Smoke Exposure and Myopia Among Children Aged 6 to 8 Years in Hong Kong. *JAMA Netw Open*. Published May 11, 2023. doi:10.1001/jamanetworkopen.2023.13006

Data
Data available: No

Additional Information
Explanation for why data not available: Data and materials supporting the results or analyses presented in our paper will be available upon reasonable request.
